# Ultrasound Viscoelastic Properties of Biomass Polysaccharide Hydrogels as Evaluated by Rheometer Equipped with Sono-Device

**DOI:** 10.3390/gels8030172

**Published:** 2022-03-09

**Authors:** Sarara Noguchi, Takaomi Kobayashi

**Affiliations:** 1 Department of Energy and Environment Engineering, Nagaoka University of Technology, 1603-1 Kamitomioka, Nagaoka 940-2188, Niigata, Japan; s123214@stn.nagaokaut.ac.jp; 2 Department of Science of Technology Innovation, Nagaoka University of Technology, 1603-1 Kamitomioka, Nagaoka 940-2188, Niigata, Japan

**Keywords:** ultrasound, hydrogels, viscoelasticity, deformation hydrogen bonds, sono-deviced rheometer

## Abstract

A viscoelastic rheometer was equipped with a sono-device and a water bath to enable measurement of storage moduli G′ and loss moduli G″ of biomass polysaccharide hydrogels such as Kanten agarose, *κ*-carrageenan, and konjac glucomannan under ultrasound (US) exposure. The action of low power of 43 kHz US on their hydrogels significantly decreased G′ of Kanten agarose and carrageenan after a few seconds of US exposure 0.1% strain. When US with 20 W output power was exposed under mechanical strain at 0.1%, lower values were obtained cyclically for 3 min US intervals. The values then reverted to the original moduli values when US was stopped in cases of Kanten agarose and carrageenan hydrogels. As G″ values were increased during US operation, the anhydro-L-galactose segments in their hydrogels were unable to relax the external US forces within the gel sufficiently, thereby leading to gel structure collapse at a higher strain percentage. These results suggest that US exposure induced deformational change in the hydrogel structure formed by hydrogen-bonded cross-links. However, US deformation was less in the case of deacetylated cross-linkage in konjac glucomannan hydrogel.

## 1. Introduction

Stark changes in properties of many kinds of intelligent materials are induced by triggered stimuli from an external source such as light, heat, or electricity [1]. These external stimuli trigger the deformation of polymeric materials. Under such triggered conditions, hydrogels particularly have been applied for medical treatments and therapies [2,3]. Among external stimuli, ultrasound (US) technology has the important features of deep penetrability from outside the body and manipulation capability with an electrical on/off switch. The application of US has been used to enhance the delivery and activity of drugs during the past two decades [4]. In this context, US trigger systems are novel and noninvasive techniques, and several ultrasonic drug-release systems using gelatinous matrices have been reported. For example, alginate hydrogels released mitoxantrone by a pulse type of US [5] to reduce tumor growth considerably. In chitosan/β-glycerophosphate hydrogels, drug release can be enhanced greatly using high-intensity focused US [6]. Chitosan/perfluorohexane nanodroplet gels show enhanced cytocompatibility under US triggering [7]. Therefore, US techniques for hydrogel matrices have been applied as stimuli and triggers for such material changes, especially for polysaccharide hydrogels [8].

For these processes, US has attracted greater attention because of its higher efficiency in the polymeric hydrogel matrix [9,10]. Nevertheless, the effects of US on hydrogels have not been adequately investigated. This is, therefore, a promising area for future research.

In medical and food-related technologies, US processes, known as sono-processes, have been applied widely in several areas of food production [11,12,13], emulsifiers [14], cosmetics [15], and medical tools [16,17]. In these areas, US applications have proven to be useful for enhancing efficiency relative to commonly applied diffusion processes on vesicles [18], micelles [19], and microbubbles [20]. Actually, US is known to be transduced in a water medium. A strong US causes polymeric chain breakage [21]. By contrast, weaker US disconnects hydrogen bonding in soft microgel polymers [22] and significantly decreases the polymeric aqueous solution viscosity [23] and polyacrylic acid–silica slurry [24] by breaking hydrogen bonds linking the polymeric chains. In addition to such aqueous polymer media, a polymeric hydrogel matrix made of cellulose [25] and chitin [26] contains large amounts of water and displays triggered properties efficiently under mild US exposure without cavitation influence, thereby indicating that US stress can destroy hydrogen bond networks in polymeric hydrogels. In such a biomass polymer, the hydrogels are safe and reliable materials, suitable for practical applications when used as a US-triggered matrix in medical and food-related fields. Therefore, ascertaining the ultrasonic responses of various biomass hydrogels is important because they have the potential to be used in applications in medicine and new development for industrialization, as in food-related areas [27].

To investigate hydrogel behaviors under US exposure, sono-device-enabled rheometry is useful to detect viscoelastic data in situ during US operation [28]. As described herein, the softening of cellulose hydrogel occurred instantaneously under US irradiation and reverted to its original viscoelastic properties when the US was ceased. In biomass cellulose and chitin hydrogels, US exposure caused gel softening in chitin and chitin–cellulose composites [26]. Nevertheless, little is known about other biomass hydrogels, especially Kanten agarose, carrageenan, and konjac glucomannan, all of which are well-known food agents.

Therefore, for the present study, a rheometer with a sono-device is applied for such hydrogels to elucidate US effects on the triggered deformation of gelatinous polysaccharides of agarose, carrageenan, and glucomannan. For Kanten agarose, *κ*-carrageenan, and konjac glucomannan, the hydrogel matrixes are known to play important roles in supporting functionality for food supplementation [29], health benefits [30], nutritional diet [31], and food additives [32]. Polysaccharides such as Kanten agarose, carrageenan, and konjac glucomannan are natural seaweed or vegetable gelatin counterparts known to form hydrogels [33,34]. The gelation of agarose and carrageenan used in these food agents is attributable to helix aggregation of anhydro-L-galactose segments. Therefore, elucidating the response of these gel properties to US is important because cellulose and chitin hydrogels have no such segments. Moreover, the US-triggered effect on other biomass polysaccharide hydrogels remains unclear at this time. The aim of the present study, using a rheometer equipped with a sono-device, is to evaluate the viscoelasticity of biomass polysaccharide hydrogels in situ during US irradiation. The results are expected to be meaningful for providing new knowledge related to biomass hydrogel properties.

## 2. Results and Discussion

### 2.1. US Effect on Biomass Hydrogel Matrix

Viscoelasticity is well known to be very useful for evaluating the polymer networking of hydrogels [35,36,37]. For the present study, representative biomass hydrogels of Kanten agarose, *κ*-carrageenan, and konjac glucomannan retained excess water in their respective matrices. The water content (WC) values were, respectively, 3384%, 3449%, and 3490% for Kanten agarose, *κ*-carrageenan, and konjac glucomannan hydrogels. These results demonstrated that the used samples all held similar amounts of water. For the viscoelastic measurements of their hydrogels, punching was 25 mm in diameter, with a thickness of 10 mm. The Kanten agarose used here was a white and semi-translucent gelatinous substance, as obtained from algae powder. Additionally, *κ*-carrageenan is a family of agarose having linear sulfated polysaccharides extracted from red edible seaweed [33] and containing sulfonic acid groups. The κ-carrageenan sample was transparent, but konjac glucomannan, the common name of the Asian plant amorphophallus konjac jelly containing glucomannan polysaccharide in the hydrogel, showed white color with turbidity after gelation. The glucomannan polysaccharides of konjac glucomannan hydrogel are partially cross-linked to form a hydrogel structure by acetylation [7,38]. 

Generally, the values of G′ or G″ moduli of hydrogels were obtained under conditions of mechanical deformation at different strain percentages and depending on the degree of strain at which the hydrogel network was broken. Our earlier research using rheometry with a sono-device indicated that cellulosic hydrogels soften under US exposure conditions because the US exposure enhanced the deformation of hydrogel [28]. However, its structural breakdown state returned to the original state when the US was turned off. Therefore, results confirmed that water desorption and hydrogen bond deformation were conducted in the presence of the US trigger.

**Figure 1:**
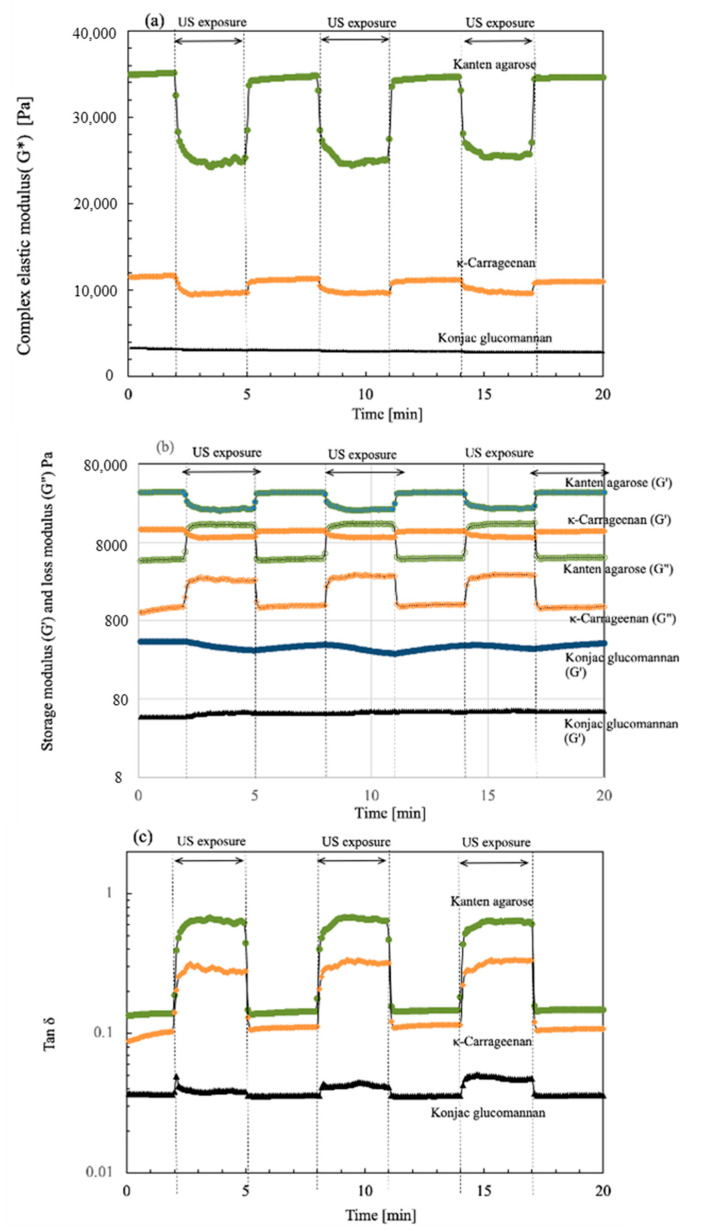
Time course of (**a**) complex modulus G*, (**b**) storage (G′) and loss modulus (G″), and (**c**) tan δ for each hydrogel in the absence and presence of US exposure at 3 min cycled intervals.

To investigate the viscoelastic change in the present biomass hydrogels in the absence and presence of US, a strain sweep was provided mechanically and was fixed at 0.1%. Then, the moduli changes were monitored when the US irradiation and non-irradiation were repeated in three cycles. Figure 1 depicts plots of the changes of (a) complex modulus G*, (b) storage moduli G′ and loss moduli G″, and (c) tan δ with elapsed time. During the detection of viscoelastic properties, the US was turned on and off at 3 min intervals. Then, the cycle was repeated for each hydrogel during 20 min operation. As shown in the time plots, the values of G* were measured for 3 min without US, as indicated at (a) at 0.1% strain. The resultant values of G* were 34,900, 11,500, and 3350 Pa, respectively, for Kanten agarose, κ-carrageenan, and konjac glucomannan. The resultant G* value indicates that the Kanten agarose was a tight hydrogel and also that konjac glucomannan was rather soft among the hydrogels used. The G* values were measured under a 0.1% strain condition, which was provided mechanically by the rheometer. The US exposure started after 3 min had passed under the 0.1% mechanical strain. For the Kanten agarose hydrogel, It was remarkable that the values of G* of 35 kPa were decreased immediately by US exposure to 20.1 kPa. They were then kept constant for the next 3 min interval up to the time the US was turned off. As US was turned off next at a 3 min interval, the G* was again returned to 35 kPa simultaneously. This cycle was repeated three times. The G* decrease and increase behavior occurs with and without US, respectively, meaning that the US softening behavior occurred cyclically. Similar behavior of the decrease or increase IN G* was observed for the carrageenan hydrogel, respectively, when the US was on and off. The initial G* value was 11.5 kPa at 0.1% strain; it then declined because of US exposure to 9100 Pa. However, the konjac glucomannan, which has a cross-linked structure at the glucomannan sites, was not observed to have cycled changes IN G* in the absence and presence of the US exposure under the 0.1% strain.

To elucidate changes in G*, the moduli components including G′ and G″ were plotted for the cyclic time intervals at 1% strain, as shown in (b). The respective values of G′ for Kanten agarose, κ-carrageenan, and konjac glucomannan were 34,600, 11,500, and 434 Pa at 0.1% strain. Those of G″ were, respectively, 4630, 1010, and 47.5 Pa at the strain %. When US was irradiated after 3 min, the G′ of Kanten agarose and κ-carrageenan decreased, but the G″ values increased and maintained this state for 3 min. This tendency for G″ to increase indicated that the propagated US force was partially absorbed by the agarose hydrogel and carrageenan hydrogel networks. It was revealed that others were repelled and pushed back without gel network relaxation. As the overall change in G′ was greater than that in G″, softening of the hydrogel resulted from the US exposure. In contrast, for konjac glucomannan, the US irradiation induced a very slight change in G′ and G″ but not a significant change. In our earlier reports describing the use of the rheometer and sono-device for cellulose [28] and chitin hydrogels [26], such an increment of change in G″ behavior under US exposure was not observed, meaning that the US irradiation decreased both G′ and G″ in cellulose and chitin, which have polysaccharides dominated by physical entanglement of the polymer chains and hydrogen-bonding networks for gelation. However, for both agarose and carrageenan hydrogels, G″ increased under US irradiation. Therefore, examining their chemical structures is a reasonable avenue of investigation. The biomass hydrogels of agarose and carrageenan have anhydro-L-galactose segments, showing a hydrophobic nature for helix aggregates [39], but cellulose and chitin have no such segments. Furthermore, in carrageenan hydrogels containing SO_3_-group, similar US-cyclic effects to those for Kanten agarose were observed for the G″ change. However, the change in G″ under US operation for the carrageenan was much less than in the agarose case. This finding might be attributable to the fact that the negatively charged groups show extended segments by electrostatic and enhanced water solvation to decrease US effects on carrageenan. Based on these comparisons, such hydrophobic anhydro-L-galactose segments have an entanglement effect on forming a partially double-helix structure [39,40]. Therefore, the US force seems to find it difficult to untangle that tangle. However, the contribution of the decrease in G′ caused by the collapse of the hydrogen bonding network linking the β-D-galactose segments strongly affects the change in the decrease in viscoelastic modulus properties of the hydrogels. Therefore, the overall gel properties caused a tendency to soften under the US exposure for agarose and carrageenan hydrogels.

As shown in Figure 1c, the value of tan δ was also cycled in the presence and absence of US exposure. Here, the values of tan δ (G″/G′) variation in the cyclic US processes meant that, as it exceeded tan δ = 1, the hydrogel became a liquid because the gel network was destroyed under US forces and mechanical strain. During US exposure, for Kanten agarose and carrageenan hydrogels, the value plots of tan δ were shifted to a lower strain percentage side, indicating that gel network deformation in each hydrogel was enhanced at lower mechanical strain. However, its value remained below tan δ = 1 for both hydrogels, with retention of gel networking. This finding indicated that the hydrogels were softened by US but remained within the range of the gel properties. Compared with Kanten agarose and κ-carrageenan, the konjac hydrogel having glucomannan segments behaved with less change in G′ and G″, along with the increase in the ultrasonic radiation force.

### 2.2. US Softening Effects under Mechanical Strain Change with Gelatinous Network

Depending upon the mechanical strain percentage of the hydrogel samples, the hydrogel structure was retained or deformed. As described in our earlier reports for cellulose [24], the gel structure was maintained up to about 1% strain. At higher deformation rates, the hydrogel structure was destroyed. Therefore, the value of G′ decreased rapidly with higher deformation rates. In its collapsed state, the input stress could not be relaxed. The G″ value tended to increase gradually.

**Figure 2:**
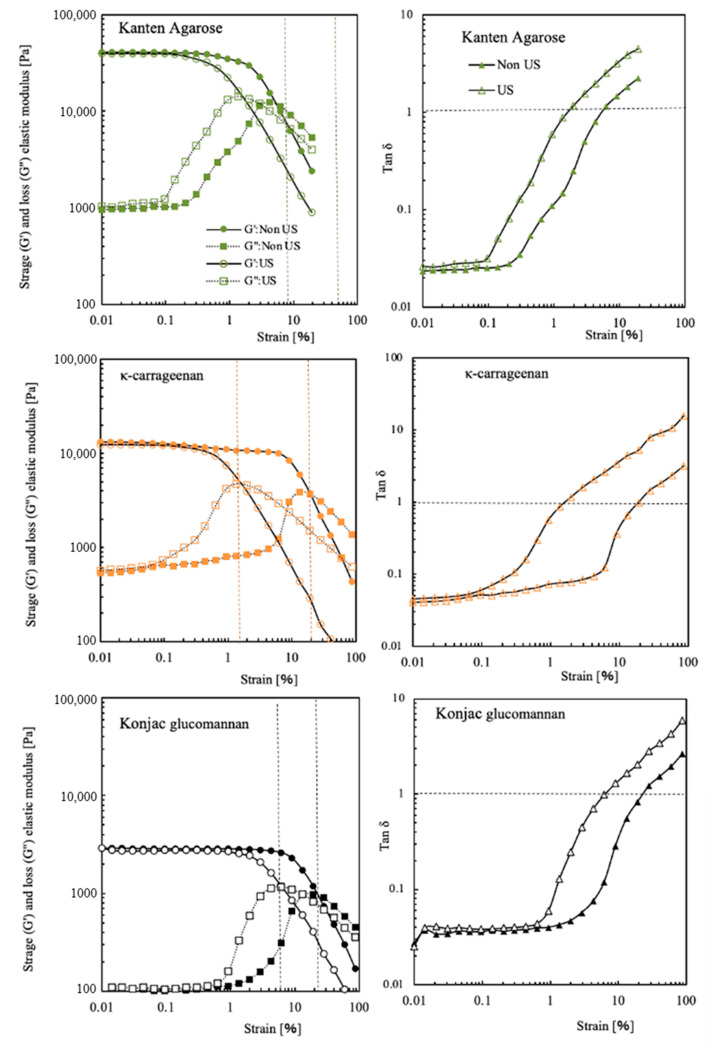
G′ and G″ moduli and tan δ of Kanten agarose, κ-carrageenan, and konjac glucomannan hydrogels at different strain% in the absence (closed symbol) and presence of US exposure (opened symbol).

For Kanten agarose, *κ*-carrageenan, and konjac glucomannan, Figure 2 shows storage and loss moduli at different mechanical strain percentages. In addition, the data obtained under US irradiation are presented as open symbols in their respective figures. The Kanten agarose hydrogel had G′ with 3.5 × 10^4^ Pa and G″ with 1.1 × 10^4^ Pa at 0.01% strain. The G′ value was constant until approximately 1% strain. Then, with increasing strain toward 100%, the G′ was decreased gradually to be 1 × 10^4^ Pa, indicating that the higher strain deformed the agarose gel structure. When the Kanten agarose hydrogel was exposed to ultrasonic radiation under the mechanical strain, the decline in G′ visibly occurred at a strain lower than 0.1%. This finding indicates strongly that US cooperated in deforming the hydrogel structure at a lower mechanical strain percentage. A similar tendency was observed in G″, as presented in open squared plots. The G″ moduli became almost constant values at about 10^3^ Pa until 0.1% strain in the absence of US. Then, the value increased to 10^4^ Pa at 5% and 1% strain, respectively, with and without US irradiation.

It is noteworthy to observe the strain percentage at which G′ and G″ are equal. For Kanten agarose hydrogel, their values of tan *δ* = 1 were observed at 6% and 1.5% strain, respectively, with and without US irradiation. When the values of G′ and G″ were equal, meaning tan *δ* = 1, in strongly cross-linked gel materials, the value of the fracture in the material or inability to follow deformation and the rigid polymer networks in the cross-linked hydrogel might not flow. At strains in excess of such strain percentages, the mechanical stress completely deforms the hydrogel matrix. However, another konjac glucomannan hydrogel showed similar phenomena of the G′ decline at 10–11% strain, even with no US exposure. Therefore, unlike agarose, these hydrogels were able to maintain their gel state even at high deformation rates. The decrease in G′ started at 0.6% strain in the carrageenan and occurred somewhat less at 2% in konjac glucomannan hydrogel when exposed to US. Actually, the lower values of G″ at 0.01% strain found for both Kanten agarose and *κ*-carrageenan indicated that gel network deformation was observed at 0.1% strain in conditions of US exposure. As shown by tan *δ* = 1, the carrageenan appeared in addition to the Kanten agarose hydrogels under US. With increasing strain percentage from 0.01% to 100%, the G″ moduli were increased at 0.1% strain, showing a maximum peak at the points of tan *δ* = 1. In contrast, the cases of konjac glucomannan hydrogel with and without US exposure, respectively, showed tan *δ* = 1 at 20% and 6% strain. This difference in the former hydrogels and the latter konjac glucomannan hydrogel is apparently attributable to the presence and absence of the covalently cross-linking structure.

**Figure 3:**
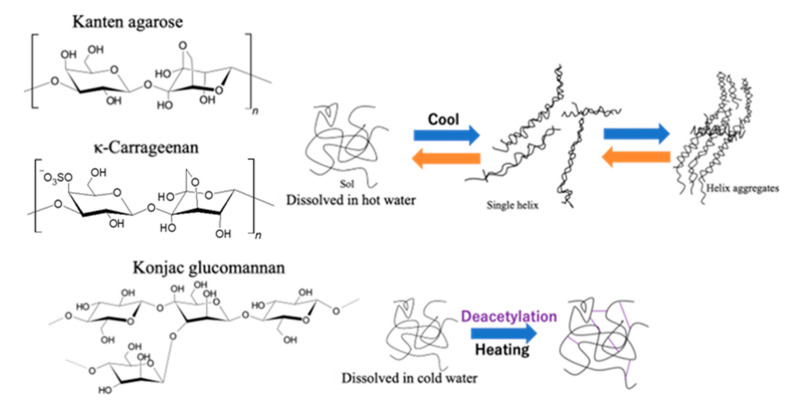
Gelation illustration of Kanten agarose, κ-carrageenan, and konjac glucomannan hydrogels.

As Figure 3 shows for illustration of gelation processes on each biomass hydrogel of Kanten agarose and *κ*-carrageenan, the hydrogel layer was composed of helix structures through the sol state of such polysaccharides [39,40]. Therefore, by the action of US-forced dispersion, ultrasonic force on a material under mechanical strain seems to form, reversibly, a sol state or single helix from hydrogels having helix aggregates. However, in the case of konjac glucomannan, gelation occurred because of the interaction of the glucomannan’s acidic moieties with alkali or neutral salt in the presence of heat to form a mesh-type gel structure [34]. This finding suggests that deacetylation cross-linked the glucomannan structure to form a hydrogel. The process retained the gelling network having tolerance under higher stress for deformation. In other words, the results in the agarose structured hydrogels indicated that both US and mechanical strain deformed the hydrogel cross-linking structure effectively to soften the matrix.

### 2.3. FT-IR Measurements of Biomass Hydrogels 

**Figure 4:**
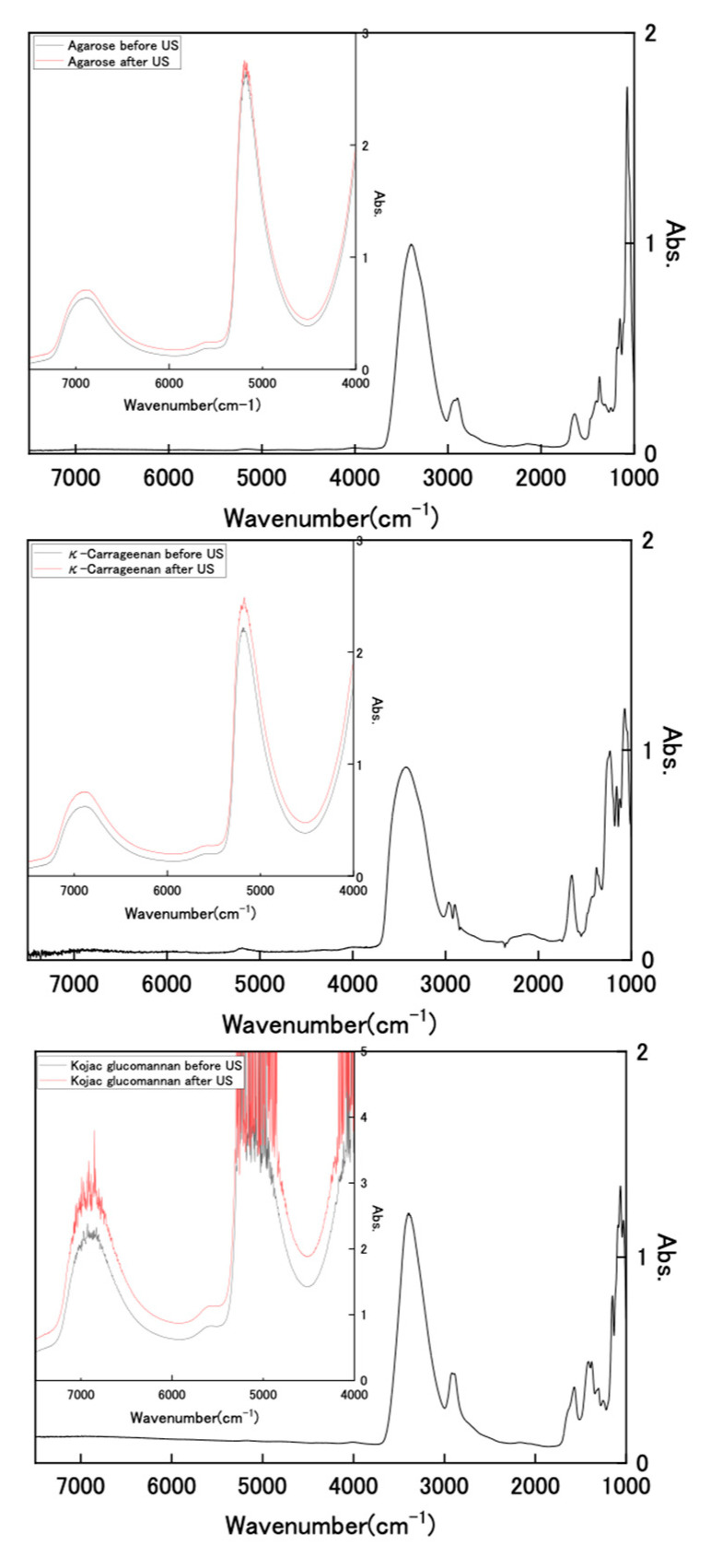
FT-IR and near-IR spectra of dried polysaccharides and wet state hydrogels with and without US exposure.

We attempted to observe the infrared (IR) spectrum of the hydrogel to analyze its composition. Figure 4 shows FT-IR and near-IR spectra of dried polysaccharides and wet state hydrogels with and without US exposure for wavenumbers of 1000–7500 cm^−1^. The FT-IR spectra of the dried samples showed clear IR bands in the range of 1000–4000 cm^−1^. However, the results for the hydrogel sample retaining water were all extremely large absorbance, with values of more than 3 in this wavenumber region. As described, the used hydrogels retained high water contents of 3384%, 3449%, and 3490%, respectively, for Kanten agarose, *κ*-carrageenan, and konjac glucomannan; the polysaccharide that was used holds about 30 times more water. Therefore, it is difficult to analyze polysaccharide components in these IR spectral measurements because of water interference. However, the hydrogel spectra show two distinct near-infrared peaks at 6800–6900 cm^−1^ and 5000 cm^−1^ in the near-IR region (inserted spectra), which are attributed, respectively, to symmetric and antisymmetric OH-stretching modes (first overtone) and the combined tone [41,42]. In cases of the observed wavenumber, the values of Kanten agarose and *κ*-carrageenan for the first overtone were 6875 and 6871 cm^−1^, respectively. These were shifted toward somewhat longer regions of 6885 cm^−1^ and 6887 cm^−1^ by US exposure. The difference between those peak shifts observed with and without US exposure was attributable to the weakening of intramolecular hydrogen bonds [43] by US exposure. The US exposure to such polysaccharide hydrogel influenced the surrounding water conditions.

## 3. Conclusions

A rheometer with a sono-device was used to measure the viscoelastic properties of hydrogels composed of agarose, carrageenan, and glucomannan during US exposure. The agarose and carrageenan hydrogels with anhydro-L-galactose and D-galactose segments were found to soften during US exposure: US promoted the gel structure collapse during large deformation. However, G″ tended to increase. The deformation force of US could not be mitigated by the gel structure composed of anhydro-L-galactose segments. By contrast, less significant US effects were observed for the konjac glucomannan hydrogel with cross-linking by deacetylated bonds.

## 4. Materials and Methods

### 4.1. Materials

**Figure 5:**
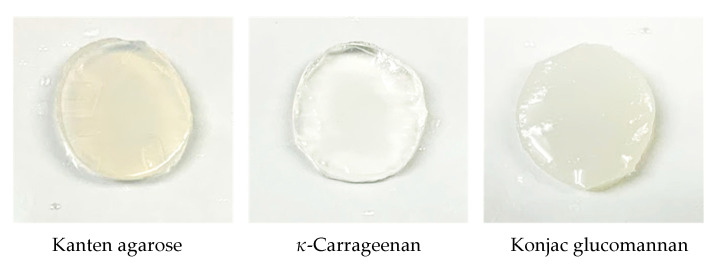
Pictures of biomass hydrogels of Kanten agar, *κ*-carrageenan, and konjac glucomannan.

Dry agarose (Kanten) purchased from Japan Garlic Co., Ltd. (Gunma, Japan) was soaked in cold water and was then boiled to dissolve the carbohydrates. Then, the aqueous solution was cooled at room temperature for gelation. Konjac glucomannan (Mac Food co. Ltd., Gunma, Japan) and the hydrogel of konjac were used after washing with excess water before use for experiments. Powders of Kanten and κ-carrageenan (Tokyo Kasei Kogyo Co. Ltd., Tokyo, Japan) were used for the following hydrogel preparation. Each 5.2 g powder was stirred to disperse it in distilled water (200 mL) at 70 °C for 2 h. After the dispersed solution was dissolved well, it was poured into the mold vessel, of which the surface was coated by Teflon. A mold containing agarose or carrageenan solution was left at rest at room temperature for 2 h. Then, the molding solution was cooled to 4 °C for 48 h during the gelation process. Thus, each hydrogel was obtained, as shown in Figure 5.

### 4.2. Rheometer Equipped with US Device

**Figure 6:**
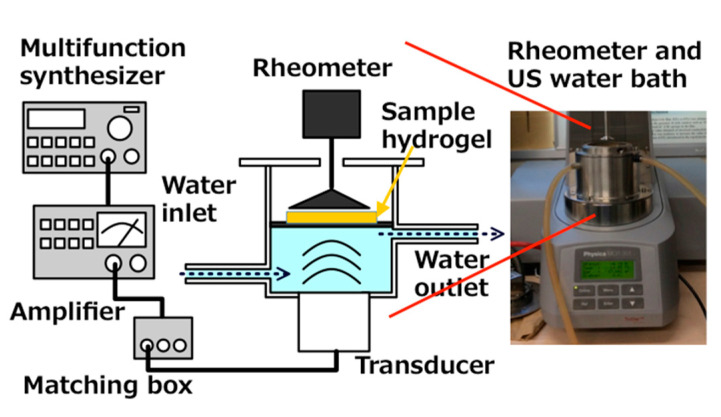
Schematic drawing US water bath for equipment to rheometer.

Viscoelasticity of hydrogels was measured (MCR 301; Anton Paar Japan, Tokyo, Japan). As Figure 6 shows, a US water bath (φ86 mm × 65 mm) was equipped with a Langevin-type transducer (HEC-45242M; Honda Electronics Co. Ltd., Aichi, Japan) at the bottom.

On the transducer, the water bath settled and a SUS 316 plate with 0.2 mm thickness was immersed in the water bath. Then, the water bath-transducer part was attached to the rheometer. To assess the hydrogel viscoelasticity, the sample was placed on a SUS 316 plate. When the US was operated at 43 kHz in the 20 W output power range and transduced to the water bath and then to the hydrogel sampler through the SUS plate simultaneously, the rheometer also produced gel deformation by a mechanical strain. Under these circumstances, G′ and G″ were measured. The emitted US power was controlled with a wave factory (WF1943B multifunction synthesizer; NF Corp., Kanagawa, Japan) at a constant of 20 W. The water was circulated to fill the water bath space under the SUS plate to maintain a constant temperature at 26 °C.

The hydrogel sample (25 mm diameter × 10 mm thickness) was placed on the SUS plate. Then, viscoelastic G′ and G″ were measured using a constant frequency of 1 Hz at room temperature or 0. 01–100% strain for deformation of the hydrogel with and without the US operation. The value of complex stress G* was calculated from $\sqrt{\left({G\prime }^{2}+{G\prime \prime }^{2}\right)}$.

**Figure 7:**
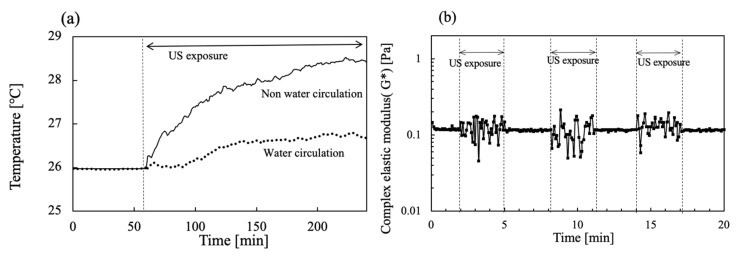
Time change in (**a**) water bath temperature in the absence and presence of water circulation and (**b**) G* in the absence and presence of US exposure for 3 min intervals.

The US device equipped with the rheometer was operated simultaneously during viscoelastic measurements. To avoid increasing the temperature, water circulation of the water bath was also conducted at 26 °C. Figure 7a shows the time change in the water bath temperature when 20 W output US power was operated for 200 s. The temperature for the water bath circulation was set at 26 °C first. However, the temperature increased concomitantly with increasing US operation time. After 200 s of US operation had passed, the water bath temperature reached about 29.4 °C. In contrast, as water in the water bath was circulated at 26 °C, a slight increase in the temperature of less than about 0.5 °C was apparent during the initial 100 s for US irradiation at 20 W. Figure 7b includes values of G* at each 3 min interval for US exposure when the viscoelasticity was operated at 1% strain without a hydrogel sample in water. The G* values were changed in the range of 0.05–0.2 Pa by the US operation. 

When the hydrogel sample was placed on the sample SUS plate on the US water bath, 5 mL water was dropped between the hydrogel and the sample stage. The rheometer was worked to rotate the rotor rod, which was placed on the hydrogel surface, with 0.01–100% of mechanical strain to the hydrogel sample. For the present study, the US was operated while mechanical deformation was occurring on the hydrogel. At the same time, the hydrogel viscoelasticity was measured. In the cases without a hydrogel sample in the water on the plate, the G* values of 0.05–0.2 Pa observed by the US operation were extremely lower than those measured for such hydrogel samples, meaning that the US noise against the viscoelastic values of the hydrogel samples can be ignored.

The right-side image is an out view of the rheometer installed with the sono-device and US water bath.

### 4.3. Evaluation of Biomass Hydrogels 

Hydrogel samples (20 mm diameter × 0.5 mm) were immersed in distilled water (300 mL) at ambient temperature for 24 h, removed, gently blotted with tissue paper to remove the excess water from the surface, and then weighed (W_2_). After the hydrogel samples were dried in a vacuum oven for 24 h, they were weighed (W_1_). The percentage water content (WC) was calculated using a dry base according to WC = ((W_2_ − W_1_)/W_1_) × 100. Fourier transform infrared spectroscopy was used to obtain FT-IR spectra of wet and dry samples (IR-prestige 21; Shimadzu Corp., Tokyo, Japan). For the wet samples, a hydrogel sample of less than about 0.4–0.7 mm thickness was put on a CaF_2_ plate (2 mm thickness; Pier Optics Co. Ltd., Gunma, Japan). Then, the other plate was pressed to cover the sample, thereby producing a thin layer between the two plates. After the CaF_2−_ plate samples were sealed with parafilm, they were exposed to US, and the spectrum was measured. The scanned wavenumber area of the FT-IR measurements was 7600 to 1000 cm^−1^ with a resolution of 8 cm^−1^, using an average of 16 scans per sample.

## Data Availability

Not applicable.
